# Fruit and vegetable intake and the risk of recurrence in patients with non-muscle invasive bladder cancer: a prospective cohort study

**DOI:** 10.1007/s10552-018-1029-9

**Published:** 2018-04-17

**Authors:** Sylvia H. J. Jochems, Frits H. M. van Osch, Raoul C. Reulen, Mitch van Hensbergen, Duncan Nekeman, Sarah Pirrie, Anke Wesselius, Frederik-Jan van Schooten, Nicholas D. James, D. Michael A. Wallace, Richard T. Bryan, K. K. Cheng, Maurice P. Zeegers

**Affiliations:** 1 Institute of Cancer and Genomic Sciences, University of Birmingham, Birmingham, UK; 2 Department of Complex Genetics and Epidemiology, School of Nutrition and Translational Research in Metabolism, Maastricht University, PO Box 616, 6200 MD Maastricht, The Netherlands; 3 Department of Public Health, Epidemiology and Biostatistics, Institute of Applied Health Research, University of Birmingham, Birmingham, UK; 4 University Hospital Birmingham, NHS Foundation Trust, Birmingham, UK

**Keywords:** Bladder cancer, Recurrence, Fruit, Vegetables, Prevention and control

## Abstract

**Introduction:**

There is some evidence that greater consumption of fruit and vegetables decreases the risk of bladder cancer. The role of fruit and vegetables in bladder cancer recurrence is still unknown.

**Objective:**

The role of total fruit and vegetable intake in relation to the risk of developing bladder cancer recurrence in a prospective cohort study.

**Methods:**

728 patients with non-muscle invasive bladder cancer (NMIBC), who completed self-administrated questionnaires on fruit and vegetable intake at time of diagnosis (over the year before diagnosis) and 1 year after diagnosis, were included. Hazard ratios and 95% confidence intervals were calculated by multivariable Cox regression for developing recurrent bladder cancer in relation to fruit and vegetable intake.

**Results:**

During 2,051 person-years of follow-up [mean (SD) follow-up 3.7 (1.5) years], 241 (33.1%) of the included 728 NMIBC patients developed a recurrence of bladder cancer. The sum of total fruit and vegetables before diagnosis was not related to a first bladder cancer recurrence (HR 1.07; 95% CI 0.78–1.47, *p* = 0.66). No association was found between greater consumption of fruit and vegetables over the year before diagnosis and the risk of developing multiple recurrences of bladder cancer (HR 1.02; 95% CI 0.90–1.15, *p* = 0.78). Among the remaining 389 NMIBC patients who reported on fruit and vegetable intake 1 year after diagnosis, no association was found between greater consumption of fruit and vegetables and a first recurrence of bladder cancer (HR 0.65; 95% CI 0.42–1.01, *p* = 0.06) nor with multiple recurrences of bladder cancer (HR 1.00, 95% CI 0.85–1.18, *p* = 1.00). Similar results were obtained when investigating the association between total intakes of fruit and vegetables separately and bladder cancer recurrence.

**Conclusion:**

Results from this study did not indicate a protective role for total fruit and vegetables in the development of a recurrence of NMIBC.

**Electronic supplementary material:**

The online version of this article (10.1007/s10552-018-1029-9) contains supplementary material, which is available to authorized users.

## Introduction

Bladder cancer is the most common malignancy of the urinary tract and is characterized by high rates of recurrence [1]. Identification of modifiable risk factors, including diet, may improve bladder cancer risk and outcome. The 2015 report of the WCRF Continuous Update Project indicated that there is limited evidence that greater consumption of vegetables and fruit decreases the risk of bladder cancer [2]. Results of a large systematic review and dose–response meta-analysis of prospective cohort studies suggested that there is little evidence that supports a beneficial effect for total fruit and vegetables against bladder cancer [3]. Although evidence on a role for fruit and vegetables in bladder cancer is limited, fruit and vegetables contain an abundant source of minerals, phytochemicals, and antioxidant nutrients with potentially anti-carcinogenic properties. Considering the low levels of antioxidants in patients with bladder cancer [4], it is conceivable that greater consumption of fruit and vegetables might result in an improved bladder cancer prognosis. A strategy to simply increase the amount of fruit and vegetables to decrease the risk of bladder cancer recurrence would be a compelling one. In a US study, Tang et al. examined the association between fruit and vegetable consumption and mortality among bladder cancer patients [5]. An inverse association between raw broccoli intake and subsequent mortality among bladder cancer patients suggested a role for vegetable intake in bladder cancer prognosis. It should be noted that only an association with raw broccoli was found, not with cooked broccoli or with other vegetables or total fruit and vegetables. No studies as of yet, have made efforts to assess fruit and vegetable intake and reduced bladder cancer recurrence. Therefore, this is the first study to investigate the role of pre-diagnosis (the year before diagnosis) and post-diagnosis (the year after diagnosis) fruit and vegetable intakes in relation to the risk of developing bladder cancer recurrence among non-muscle invasive bladder cancer (NMIBC) patients.

## Methods

### The Bladder Cancer Prognosis Programme

This study is part of the Bladder Cancer Prognosis Programme (BCPP), a prospective cohort study in the West Midlands region of England. Details of the cohort have been published previously [6]. Briefly, during the enrolment period (December 2005–October 2011), a total of 1,550 male and female patients (age ≥ 18 years) were enrolled based on abnormal cystoscopic findings suggestive of bladder cancer. Case report forms (CRFs) contained data on tumor characteristics, initial treatment, and event data including recurrences. Bladder cancer recurrence was defined as the new occurrence of a NMIBC (stage Ta, T1, or pTis) at the same or at a different site as the initial primary bladder tumor and excluding recurrence identified at the first check cystoscopy. Written informed consent was obtained from all participants. The study protocol was approved by the Nottingham Research Ethics Committee (06/MRE04/65) and registered on ClinicalTrials.gov (NCT00553566).

### Data collection

Data on social support, health-related quality of life, socio-demographics, medical history, and health-related behaviors including dietary intake were collected by a trained research nurse using semi-structured face-to-face interviews and use of a questionnaire around time of diagnosis. Patients were asked about habitual dietary intake over the previous year before diagnosis. The developed version of the food-frequency part of the questionnaire aims to assess the dietary intake, by asking the participants to report the frequency of consumption of approximately 16-line items over the last year. The frequency of fruit and vegetables asked in the questionnaire consisted of six categories: never or less than once per month, 1–3 times per month, once a week, 2–4 times per week, 5–6 times per week, or at least once per day. The research nurse informed the patients that one portion of vegetables or fruit is about 80 g. Total fruit and vegetable intakes were computed as the sum of servings of all of groups of fruit and vegetables: citrus fruit, stone fruit, fleshy fruit, soft fruit, and vine fruit. Vegetable subgroups included: flower vegetables, leafy vegetables, stem vegetables, fruit vegetables, mushrooms, bulbs, and roots. Repeated fruit and vegetable intakes were collected through postal follow-up questionnaires 1–5 year after diagnosis. This study focuses only on fruit and vegetable intakes over the year before and the year after diagnosis.

### Exclusion criteria

A total of 244 participants with no evidence of a bladder tumor (T0), patients who had a tumor that could not be assessed (Tx) (*n* = 116), had muscle invasive bladder cancer (MIBC) (*n* = 275), had radiotherapy (on suspicion of MIBC) (*n* = 8), received no transurethral resection of the primary bladder tumor (TURBT) (*n* = 16), had incomplete data on tumor stage, grade, size, and multiplicity (*n* = 53) or smoking (*n* = 94), and missing data on fruit and vegetable intake before diagnosis (*n* = 16), were excluded from this study. The final analyses for fruit and vegetable intakes and bladder cancer recurrence comprised 728 NMIBC patients.

Almost half of the remaining 728 patients did not complete a follow-up questionnaire 1 year after diagnosis or developed a recurrence before completing the follow-up questionnaire (*n* = 339). A total of 389 patients remained for investigating the association between fruit and vegetable intake over the year after diagnosis and bladder cancer recurrence analyses.

### Statistical analysis

A χ^2^ test was used to compare categorical data between patients with and without bladder cancer recurrences. Patients became at risk from the date of first TURBT and remained at risk until the earliest occurrence of: a recurrence of bladder cancer, cystectomy, death, the most recent surveillance cystoscopy, or study end (5-year post-TURBT). To identify possibly influential outliers in total fruit and vegetable intake, Cook’s Distance was used. Cox regression was used to calculate hazard ratios (HRs) and 95% confidence intervals (95% CI) of developing recurrent bladder cancer in relation to the sum of total fruit and vegetables, and total fruit and total vegetables separately. Additionally, associations between each subgroup of fruit (citrus/stone/fleshy/soft/vine) and each subgroup of vegetables (flower/leaf/stem/fruit vegetables/mushrooms/bulbs/roots) were examined with bladder cancer recurrence.

To investigate the risk of fruit and vegetables with multiple recurrences, a conditional risk set model measuring time from the previous bladder cancer recurrence was used. A conditional risk set model measuring time from the previous event was used to analyze time to multiple recurrences of bladder cancer. The proportional hazards assumption was tested using Schoenfeld residuals [7]. Patients were divided into three categories: the first category for total fruit intake corresponded to < 1 portion a day, the second category to 1–1.5 portions a day, and the third category to > 1.5 portions a day. For total vegetables, the categories corresponded with < 1.5 portion a day, 1.5–2.5 portions a day, and > 2.5 portions a day. For the sum of fruit and vegetables, the categories corresponded with < 2.5 portions a day, 2.5–4 portions a day, and > 4 portions a day. The association between total fruit and vegetable intake and bladder cancer recurrence was examined in both crude models and multivariate models. Confounders were considered a priori as factors known or suspected to be related with cancer recurrence and/or fruit and vegetables and were included in the multivariate regression models. Confounders included: age at diagnosis (continuous) [8], sex (male/female) [9], smoking status (never/former/current smoker) [10], and tumor characteristics including stage (pTa/pTis/pT1), grade (1/2/3), size of largest tumor (< 3 mm/≥ 3 mm), and tumor multiplicity (1/> 1) [11–13]. Multivariate time to multiple recurrences regression models were additionally corrected for re-resection of a bladder tumor as second-line treatment (yes/no). *p* values were 2-sided with a significance level of 0.05. All analyses were performed using Stata software version 14.

## Results

During 2,051 person-years of follow-up [mean (SD) follow-up 3.7 (1.5) years], 241 (33.1%) of the 728 NMIBC patients developed one or more recurrences of bladder cancer. A total of 84 (34.9%) of the 241 patients developed a second recurrence, 36 (14.9%) a third recurrence, 18 (7.5%) a fourth recurrence, 6 (2.5%) a fifth recurrence, 3 (1.2%) a sixth recurrence, 2 (0.8%) a seventh recurrence, and 1 (0.4%) an eighth recurrence. Table 1 presents the characteristics of the patients at diagnosis and initial treatment. Patients were mainly male (80%), Caucasian (97%), and former or current smokers (36 and 50%, respectively). Mean age at diagnosis was 69 years. The average intake of fruits and vegetables was evenly distributed between patients who had a recurrence of bladder cancer and those who had not (mean total fruit and vegetable intake for all patients 3,5 portions a day—283 and 275 g/day, respectively). No outliers were detected in fruit and vegetable intakes.

**Table 1:** Patient characteristics at diagnosis for 728 NMIBC patients treated with transurethral resection of a primary bladder tumor

	Number at risk (%)
Sex	
Male	584 (80%)
Female	144 (20%)
Age at diagnosis (years)	
< 45	15 (2%)
45–64	211 (29%)
65–84	461 (63%)
≥ 85	41 (6%)
Smoking status	
Current smokers	366 (50%)
Former smokers	263 (36%)
Never smokers	99 (14%)
Alcohol consumption	
Drinkers	545 (75%)
Non-drinkers	183 (25%)
Educational level	
High	76 (10%)
Middle	106 (15%)
None/low	165 (23%)
BCG intravesical immunotherapy	
Yes	112 (15%)
No	281 (39%)
Mitomycin C intravesical chemotherapy	
Yes	342 (47%)
No	72 (10%)
Tumor stage	
pTa	486 (67%)
pT1	236 (32%)
pTis	6 (1%)
Tumor grade	
1	214 (29%)
2	263 (36%)
3	251 (35%)
Size largest tumor (cm)	
< 3	460 (63%)
≥ 3	268 (37%)
Tumors multiplicity	
1	432 (59%)
> 1	296 (41%)
No of recurrences over 5 years	
0	487 (67%)
1	157 (22%)
> 1	84 (11%)

where the data contain missing values the percentages do not add up to 100%

Table 2 presents HRs with corresponding 95% CI for pre- and post-diagnosis total fruit and vegetable intakes and time to first and time to multiple recurrences of bladder cancer. The sum of total fruit and vegetable intake before diagnosis did not affect the development of a first recurrence of bladder cancer (HR 1.07; 95% CI 0.78–1.47, *p* = 0.66) nor multiple recurrences (HR 1.02; 95% CI 0.90–1.15, *p* = 0.78). Total vegetable intake was not associated with a first recurrence or bladder cancer (HR 1.02, 95% CI 0.74–1.41, *p* = 0.88)—similar results were found for the association with multiple recurrences (HR 0.97; 95% CI 0.86–1.11, *p* = 0.69). Total fruit intake before diagnosis was not associated with a first recurrence of bladder cancer (HR 0.85; 95% CI 0.63–1.14, *p* = 0.29). Results for total fruit intake and multiple recurrences indicated also no association with multiple recurrences (HR 1.05; 95% CI 0.91–1.20).

**Table 2:** Hazard ratios (HR) and 95% confidence intervals (95% CI) for Cox proportional hazard models predicting recurrence of bladder cancer, based on total fruit, total vegetable, and total fruit + vegetable intake over the year before diagnosis, in 728 NMIBC patients

	*n*	Events	Model 1^a^		Model 2^b^		Model 3^c^		*p* for trend
			HR (95% CI)	*p* value	HR (95% CI)	*p* value	HR (95% CI)	*p* value	
Time to first recurrence									
Total fruit + vegetables									
T1	225	69	1.00 (1.00–1.00)		1.00 (1.00–1.00)		1.00 (1.00–1.00)		0.68
T2	251	82	1.07 (0.78–1.46)	0.69	1.08 (0.79–1.48)	0.63	1.09 (0.79–1.50)	0.60	
T3	252	90	1.15 (0.84–1.57)	0.38	1.14 (0.84–1.56)	0.40	1.07 (0.78–1.47)	0.66	
Total vegetables									
T1	216	67	1.00 (1.00–1.00)		1.00 (1.00–1.00)		1.00 (1.00–1.00)		0.86
T2	259	84	0.98 (0.72–1.35)	0.91	1.00 (0.73–1.37)	1.00	0.97 (0.70–1.33)	0.84	
T3	253	90	1.11 (0.81–1.52)	0.52	1.11 (0.81–1.52)	0.53	1.02 (0.74–1.41)	0.88	
Total fruit									
T1	295	99	1.00 (1.00–1.00)		1.00 (1.00–1.00)		1.00 (1.00–1.00)		0.30
T2	163	61	1.20 (0.87–1.66)	0.26	1.21 (0.88–1.67)	0.25	1.22 (0.89–1.69)	0.22	
T3	270	81	0.85 (0.63–1.14)	0.27	0.84 (0.63–1.13)	0.25	0.85 (0.63–1.14)	0.29	
Time to multiple recurrences									
Total fruit + vegetables									
T1	225	102	1.00 (1.00–1.00)		1.00 (1.00–1.00)		1.00 (1.00–1.00)		0.76
T2	251	140	1.03 (0.91–1.15)	0.66	1.01 (0.90–1.14)	0.87	1.00 (0.88–1.12)	0.94	
T3	252	149	1.03 (0.91–1.16)	0.64	1.02 (0.90–1.16)	0.73	1.02 (0.90–1.15)	0.78	
Total vegetables									
T1	216	105	1.00 (1.00–1.00)		1.00 (1.00–1.00)		1.00 (1.00–1.00)		0.77
T2	259	130	0.99 (0.87–1.13)	0.89	0.99 (0.87–1.13)	0.85	0.95 (0.83–1.08)	0.44	
T3	253	156	1.00 (0.88–1.13)	0.94	1.00 (0.88–1.13)	0.94	0.97 (0.86–1.11)	0.69	
Total fruit									
T1	295	145	1.00 (1.00–1.00)		1.00 (1.00–1.00)		1.00 (1.00–1.00)		0.50
T2	163	119	1.06 (0.94–1.19)	0.34	1.06 (0.94–1.19)	0.34	1.06 (0.94–1.21)	0.32	
T3	270	127	1.04 (0.92–1.17)	0.58	1.03 (0.89–1.18)	0.71	1.05 (0.91–1.20)	0.52	

^a^Model 1 is unadjusted

^b^Model 2 is adjusted for age, sex, and smoking status

^c^Model 3 is adjusted for age, sex, smoking status, tumor stage, grade, size, and multiplicity (and additionally adjusted for re-resection of a bladder tumor (second transurethral resection) in the time to multiple recurrences analysis)

Table 3 presents the results for the remaining 389 NMIBC patients on total fruit and vegetable intake after diagnosis—no associations were observed between the sum of total fruit and vegetable intake and a first recurrence (HR 0.65; 95% CI 0.42–1.01, *p* = 0.06) or multiple recurrences of bladder cancer (HR 1.00; 95% CI 0.85–1.18, *p* = 1.00). Total vegetable intake after diagnosis did not affect a first bladder cancer recurrence (HR 0.77; 95% CI 0.50–1.18, *p* = 0.23) nor multiple recurrences (HR 0.96; 95% CI 0.74–1.09, *p* = 0.61). Post-diagnosis intakes of total fruit were also not related to bladder cancer recurrence (HR 0.65; 95% CI 0.44–1.00, *p* = 0.05 for a first recurrence and HR 1.02; 0.87–1.20, *p* = 0.79 for multiple recurrences). In addition, results of the Cox proportional hazard models predicting the development of a first and multiple recurrences of bladder cancer, based on subgroups of fruits and vegetables and vitamin supplement use, can be found in the online supplementary file (Tables S1–S12). Analyses on the consumption of subgroups of fruits and vegetables and vitamin supplement use showed no association with developing a recurrence of bladder cancer.

**Table 3:** Hazard ratios (HR) and 95% confidence intervals (95% CI) for Cox proportional hazard models predicting recurrence of bladder cancer, based on total fruit, total vegetable, and total fruit + vegetable intake over the year after diagnosis, in 389 NMIBC patients

	*n*	Events	Model 1^a^		Model 2^b^		Model 3^c^		*p* for trend
			HR (95% CI)	*p* value	HR (95% CI)	*p* value	HR (95% CI)	*p* value	
Time to first recurrence									
Total fruit + vegetables									
T1	138	54	1.00 (1.00–1.00)		1.00 (1.00–1.00)		1.00 (1.00–1.00)		0.06
T2	136	55	1.00 (0.69–1.46)	1.00	0.99 (0.68–1.44)	0.94	0.93 (0.62–1.38)	0.72	
T3	115	35	0.68 (0.45–1.04)	0.08	0.70 (0.46–1.07)	0.10	0.65 (0.42–1.01)	0.06	
Total vegetables									
T1	133	51	1.00 (1.00–1.00)		1.00 (1.00–1.00)		1.00 (1.00–1.00)		0.23
T2	144	54	0.93 (0.64–1.36)	0.72	0.94 (0.64–1.38)	0.75	0.84 (0.57–1.26)	0.40	
T3	112	39	0.83 (0.55–1.25)	0.37	0.86 (0.56–1.31)	0.48	0.77 (0.50–1.18)	0.23	
Total fruit									
T1	151	62	1.00 (1.00–1.00)		1.00 (1.00–1.00)		1.00 (1.00–1.00)		0.05
T2	103	41	0.93 (0.63–1.39)	0.74	0.93 (0.62–1.38)	0.70	0.96 (0.63–1.44)	0.83	
T3	135	41	0.67 (0.45–0.99)	0.04	0.67 (0.45–0.99)	0.04	0.65 (0.44–1.00)	0.05	
Time to multiple recurrences									
Total fruit + vegetables									
T1	138	89	1.00 (1.00–1.00)		1.00 (1.00–1.00)		1.00 (1.00–1.00)		0.96
T2	136	82	0.94 (0.82–1.08)	0.41	0.96 (0.84–1.10)	0.57	0.98 (0.86–1.13)	0.79	
T3	115	50	0.96 (0.80–1.15)	0.66	0.97 (0.81–1.17)	0.77	1.00 (0.85–1.18)	1.00	
Total vegetables									
T1	133	77	1.00 (1.00–1.00)		1.00 (1.00–1.00)		1.00 (1.00–1.00)		0.58
T2	144	91	0.96 (0.84–1.10)	0.55	0.97 (0.84–1.11)	0.66	0.97 (0.82–1.09)	0.63	
T3	112	53	0.94 (0.79–1.11)	0.44	0.95 (0.80–1.12)	0.53	0.96 (0.74–1.09)	0.61	
Total fruit									
T1	151	99	1.00 (1.00–1.00)		1.00 (1.00–1.00)		1.00 (1.00–1.00)		0.96
T2	103	66	0.91 (0.80–1.04)	0.18	0.93 (0.81–1.06)	0.25	0.93 (0.81–1.06)	0.28	
T3	135	56	0.98 (0.83–1.16)	0.79	0.99 (0.84–1.18)	0.93	1.02 (0.87–1.20)	0.79	

^a^Model 1 is unadjusted

^b^Model 2 is adjusted for age, sex, and smoking status

^c^Model 3 is adjusted for age, sex, smoking status, tumor stage, grade, size, and multiplicity (and additionally adjusted for re-resection of a bladder tumor (second transurethral resection) in the time to multiple recurrences analysis)

## Discussion

This is the first study to investigate the role of fruit and vegetable intakes before and after diagnosis and the recurrence of NMIBC. No evidence of an association was found between intakes of fruits and vegetables and bladder cancer recurrence in this prospective cohort study. It seems plausible, however, that substances in fruit and vegetables including minerals, phytochemicals, and antioxidant nutrients could protect against the development of cancer by reducing oxidative stress and DNA damage caused by free radicals. Furthermore, it has been suggested that substances in fruit and vegetables could affect pathways involved in inflammation and cell proliferation and apoptosis. More specifically, when glucosinolates found in cruciferous vegetables are broken down to form biologically active compounds including isothiocyanates and indoles, they have shown to inhibit the development of bladder cancer in mice and rats. Considering the low levels of antioxidants in patients with bladder cancer [4], it is conceivable that higher intakes of fruits and vegetables might result in an improved bladder cancer prognosis as well. An explanation for the lack of an association between fruit and vegetable intake and bladder cancer recurrence could be the difficulty to pinpoint the effect of one food group like fruits and vegetables given such a convoluted situation. Our diet consists of many different food items comprising many different nutrients, all working together at the same time. It seems plausible that any preventive effect may result from a combination of influences on pathways involved in the development of bladder cancer. Another explanation could be that tumor recurrence is more influenced by field cancerisation and tumor biology than beneficial bioactive compounds in fruit and vegetables [14, 15].

It is inevitable that usual fruit and vegetable intakes were not measured without error—recall bias and measurement errors in dietary intake cannot be excluded and are a common limitation of epidemiological studies. Sample sizes per average use category for subgroups of fruits and vegetables were small, especially in the post-diagnosis intake analyses. It is reasonable to assume that these small sample sizes have resulted in limited statistical power to examine associations between subgroups of fruits and vegetables and recurrence accurately. Finally, FFQs may be a source of error due to inadequate food composition information, a limited food list, reporting average intake over a long period of time, under- or over-reporting of intake, and social desirability bias. Hence, the null results of this study could indicate that the use of FFQs may not be adequate to detect associations of total fruit and vegetable intake with bladder cancer recurrence, even if a relationship exists.

## Conclusion

In this prospective cohort study, a lack of a protective association between the intake of fruit and vegetables and the risk of developing bladder cancer recurrence was demonstrated. To this extend, no recommendations to increase the amount of fruits and vegetables after a diagnosis of NMIBC to prevent bladder cancer recurrence can be provided.

## Electronic supplementary material

Below is the link to the electronic supplementary material.

Online Supplemental Tables S1–S8. Hazard ratios (HR) and 95% confidence intervals (95% CI) for Cox proportional hazard models predicting recurrence of bladder cancer, based on subgroups of fruits and vegetables and vitamin supplements consumed in the year before and in the year after diagnosis. (DOCX 121 KB)
