# Supplementary material for: Fruit and vegetable intake and the risk of recurrence in patients with non-muscle invasive bladder cancer: a prospective cohort study
Source: Cancer Causes Control. 2018 Apr 17;29(6):573–9. doi: 10.1007/s10552-018-1029-9 (PMC5938309; doi:10.1007/s10552-018-1029-9)
Supplement: Supplementary file 1 — Online Supplemental Tables S1–S8. Hazard ratios (HR) and 95% confidence intervals (95% CI) for Cox proportional hazard models predicting recurrence of bladder cancer, based on subgroups of fruits and vegetables and vitamin supplements consumed in the year before and in the year after diagnosis. (DOCX 121 KB) [file 10552_2018_1029_MOESM1_ESM.docx]

**SUPPLEMENTARY DATA ‘Fruit and vegetable intake and the risk of recurrence in patients with non-muscle invasive bladder cancer: a prospective cohort study**

**Pre-diagnosis analysis**

| **Table 1. Hazard ratios (95% confidence intervals) of pre-diagnosis vegetable intake and a first bladder cancer recurrence (n=728)** | | | | | | | | | |
| --- | --- | --- | --- | --- | --- | --- | --- | --- | --- |
|  |  |  | **Model 1*** |  | **Model 2**** |  | **Model 3***** |  |  |
|  | **n** | **events** | **HR (95% CI)** | **p-value** | **HR (95% CI)** | **p-value** | **HR (95% CI)** | **p-value** | **p trend** |
| **Fruit vegetables (e.g. tomato, cucumber, aubergine)** |  |  |  |  |  |  |  |  |  |
| Never or <1 per month | 44 | 17 | 1.00 (ref.) | . | 1.00 (ref.) | . | 1.00 (ref.) | . | 0.97 |
| 1-3 per month | 45 | 12 | 0.58 (0.28-1.19) | 0.13 | 0.57 (0.27-1.17) | 0.13 | 0.54 (0.26-1.11) | 0.09 |  |
| Once a week | 99 | 29 | 0.70 (0.39-1.25) | 0.23 | 0.71 (0.39-1.28) | 0.25 | 0.62 (0.34-1.12) | 0.11 |  |
| 2-4 per week | 269 | 88 | 0.75 (0.45-1.23) | 0.25 | 0.74 (0.45-1.24) | 0.25 | 0.66 (0.39-1.09) | 0.11 |  |
| 5-6 per week | 99 | 29 | 0.62 (0.34-1.12) | 0.11 | 0.60 (0.33-1.09) | 0.09 | 0.53 (0.29-0.97) | 0.04 |  |
| At least once per day | 172 | 66 | 0.90 (0.54-1.52) | 0.70 | 0.92 (0.54-1.57) | 0.75 | 0.77 (0.45-1.32) | 0.34 |  |
| **Flower vegetables (e.g. broccoli, cauliflower)** |  |  |  |  |  |  |  |  |  |
| Never or <1 per month | 68 | 24 | 1.00 (1.00-1.00) | . | 1.00 (1.00-1.00) | . | 1.00 (1.00-1.00) | . | 0.51 |
| 1-3 per month | 60 | 18 | 0.83 (0.45-1.52) | 0.54 | 0.80 (0.44-1.48) | 0.48 | 0.76 (0.41-1.40) | 0.38 |  |
| Once a week | 172 | 52 | 0.77 (0.48-1.24) | 0.28 | 0.78 (0.48-1.26) | 0.31 | 0.76 (0.48-1.20) | 0.23 |  |
| 2-4 per week | 323 | 99 | 0.80 (0.52-1.24) | 0.31 | 0.79 (0.51-1.22) | 0.28 | 0.74 (0.49-1.13) | 0.17 |  |
| 5-6 per week | 62 | 27 | 1.18 (0.68-2.04) | 0.56 | 1.12 (0.64-1.95) | 0.69 | 1.04 (0.61-1.79) | 0.88 |  |
| At least once per day | 43 | 21 | 1.52 (0.84-2.75) | 0.16 | 1.46 (0.80-2.65) | 0.22 | 1.19 (0.65-2.20) | 0.57 |  |
| **Leafy vegetables (e.g. spinach, cabbage, lettuce)** |  |  |  |  |  |  |  |  |  |
| Never or <1 per month | 67 | 20 | 1.00 (1.00-1.00) | . | 1.00 (1.00-1.00) | . | 1.00 (1.00-1.00) | . | 0.63 |
| 1-3 per month | 56 | 18 | 0.97 (0.52-1.82) | 0.92 | 0.96 (0.51-1.81) | 0.90 | 0.94 (0.49-1.80) | 0.85 |  |
| Once a week | 189 | 69 | 1.13 (0.70-1.85) | 0.61 | 1.12 (0.69-1.83) | 0.65 | 1.12 (0.67-1.87) | 0.65 |  |
| 2-4 per week | 312 | 94 | 0.90 (0.56-1.45) | 0.67 | 0.91 (0.56-1.46) | 0.68 | 0.87 (0.53-1.43) | 0.59 |  |
| 5-6 per week | 60 | 26 | 1.36 (0.76-2.44) | 0.31 | 1.34 (0.74-2.41) | 0.33 | 1.28 (0.70-2.32) | 0.42 |  |
| At least once per day | 44 | 14 | 0.92 (0.47-1.81) | 0.81 | 0.87 (0.44-1.73) | 0.70 | 0.79 (0.39-1.60) | 0.51 |  |
| **Stem vegetables (e.g. asparagus, celery, fennel)** |  |  |  |  |  |  |  |  |  |
| Never or <1 per month | 369 | 124 | 1.00 (1.00-1.00) | . | 1.00 (1.00-1.00) | . | 1.00 (1.00-1.00) | . | 0.91 |
| 1-3 per month | 169 | 52 | 0.86 (0.62-1.20) | 0.38 | 0.89 (0.64-1.23) | 0.48 | 0.84 (0.60-1.17) | 0.30 |  |
| Once a week | 105 | 30 | 0.81 (0.54-1.21) | 0.30 | 0.81 (0.55-1.20) | 0.30 | 0.79 (0.52-1.18) | 0.25 |  |
| 2-4 per week | 69 | 29 | 1.23 (0.83-1.82) | 0.31 | 1.19 (0.81-1.77) | 0.38 | 1.13 (0.74-1.71) | 0.57 |  |
| 5-6 per week | 6 | 3 | 1.79 (0.48-6.64) | 0.39 | 1.70 (0.45-6.44) | 0.44 | 1.85 (0.56-6.10) | 0.31 |  |
| At least once per day | 10 | 3 | 0.91 (0.26-3.23) | 0.89 | 0.86 (0.24-3.13) | 0.82 | 0.73 (0.19-2.84) | 0.65 |  |
| **Mushrooms** |  |  |  |  |  |  |  |  |  |
| Never or <1 per month | 177 | 57 | 1.00 (1.00-1.00) | . | 1.00 (1.00-1.00) | . | 1.00 (1.00-1.00) | . | 0.90 |
| 1-3 per month | 184 | 63 | 1.05 (0.74-1.49) | 0.79 | 1.05 (0.73-1.50) | 0.79 | 1.06 (0.74-1.51) | 0.75 |  |
| Once a week | 206 | 67 | 0.96 (0.68-1.36) | 0.82 | 0.99 (0.70-1.41) | 0.97 | 0.93 (0.65-1.34) | 0.71 |  |
| 2-4 per week | 143 | 47 | 1.00 (0.68-1.48) | 0.99 | 1.04 (0.70-1.53) | 0.85 | 1.01 (0.68-1.49) | 0.97 |  |
| 5-6 per week | 7 | 3 | 1.52 (0.39-5.89) | 0.55 | 1.67 (0.44-6.28) | 0.45 | 1.82 (0.47-7.03) | 0.39 |  |
| At least once per day | 11 | 4 | 1.49 (0.50-4.50) | 0.48 | 1.48 (0.49-4.51) | 0.49 | 1.23 (0.41-3.63) | 0.71 |  |

*Model 1 was unadjusted, **Model 2 was adjusted for age, sex, and smoking status, ***Model 3 was adjusted for age, sex, smoking status, and tumour stage, grade, size and multiplicity.

| **Table 1. (continued)** | | | | | | | | | |
| --- | --- | --- | --- | --- | --- | --- | --- | --- | --- |
|  |  |  | **Model 1*** |  | **Model 2**** |  | **Model 3***** |  |  |
|  | **n** | **events** | **HR (95% CI)** | **p-value** | **HR (95% CI)** | **p-value** | **HR (95% CI)** | **p-value** | **p trend** |
| **Bulbs (e.g. onion, garlic, leek, shallot)** |  |  |  |  |  |  |  |  |  |
| Never or <1 per month | 121 | 40 | 1.00 (1.00-1.00) | . | 1.00 (1.00-1.00) | . | 1.00 (1.00-1.00) | . | 0.32 |
| 1-3 per month | 103 | 36 | 1.03 (0.66-1.60) | 0.89 | 1.04 (0.67-1.61) | 0.87 | 0.95 (0.61-1.47) | 0.82 |  |
| Once a week | 170 | 62 | 1.10 (0.74-1.63) | 0.63 | 1.16 (0.78-1.73) | 0.45 | 1.03 (0.69-1.55) | 0.87 |  |
| 2-4 per week | 241 | 75 | 0.88 (0.60-1.29) | 0.50 | 0.90 (0.61-1.32) | 0.59 | 0.83 (0.56-1.22) | 0.34 |  |
| 5-6 per week | 59 | 18 | 0.89 (0.50-1.57) | 0.69 | 0.97 (0.55-1.72) | 0.92 | 0.90 (0.51-1.59) | 0.72 |  |
| At least once per day | 34 | 10 | 0.88 (0.43-1.79) | 0.72 | 0.97 (0.47-1.98) | 0.93 | 0.82 (0.40-1.69) | 0.59 |  |
| **Roots (e.g. beetroot, swede, carrot, parsnip)** |  |  |  |  |  |  |  |  |  |
| Never or <1 per month | 69 | 25 | 1.00 (1.00-1.00) | . | 1.00 (1.00-1.00) | . | 1.00 (1.00-1.00) | . | 0.93 |
| 1-3 per month | 71 | 24 | 0.87 (0.49-1.54) | 0.63 | 0.84 (0.47-1.49) | 0.55 | 0.82 (0.47-1.44) | 0.49 |  |
| Once a week | 173 | 49 | 0.63 (0.39-1.03) | 0.06 | 0.61 (0.38-0.99) | 0.04 | 0.58 (0.36-0.95) | 0.03 |  |
| 2-4 per week | 304 | 95 | 0.76 (0.48-1.19) | 0.23 | 0.73 (0.46-1.15) | 0.17 | 0.69 (0.43-1.09) | 0.11 |  |
| 5-6 per week | 68 | 29 | 1.05 (0.61-1.78) | 0.87 | 0.99 (0.58-1.70) | 0.98 | 0.84 (0.49-1.44) | 0.52 |  |
| At least once per day | 43 | 19 | 1.10 (0.60-2.00) | 0.75 | 0.99 (0.53-1.83) | 0.96 | 0.93 (0.50-1.75) | 0.82 |  |

*Model 1 was unadjusted, **Model 2 was adjusted for age, sex, and smoking status, ***Model 3 was adjusted for age, sex, smoking status, and tumour stage, grade, size and multiplicity.

| **Table 2. Hazard ratios (95% confidence intervals) of pre-diagnosis fruit intake and a first bladder cancer recurrence (n=728)** | | | | | | | | | |
| --- | --- | --- | --- | --- | --- | --- | --- | --- | --- |
|  |  |  |  |  |  |  |  |  |  |
|  |  |  | **Model 1*** |  | **Model 2**** |  | **Model 3***** |  |  |
|  | **n** | **events** | **HR (95% CI)** | **p-value** | **HR (95% CI)** | **p-value** | **HR (95% CI)** | **p-value** | **p trend** |
| **Citrus fruits (e.g. orange, lemon, lime, grapefruit)** |  |  |  |  |  |  |  |  |  |
| Never or <1 per month | 223 | 66 | 1.00 (1.00-1.00) | . | 1.00 (1.00-1.00) | . | 1.00 (1.00-1.00) | . | 0.77 |
| 1-3 per month | 109 | 42 | 1.28 (0.88-1.87) | 0.20 | 1.35 (0.92-1.98) | 0.13 | 1.34 (0.91-1.98) | 0.14 |  |
| Once a week | 107 | 46 | 1.59 (1.10-2.31) | 0.01 | 1.69 (1.16-2.47) | 0.01 | 1.72 (1.16-2.53) | 0.01 |  |
| 2-4 per week | 155 | 44 | 0.93 (0.63-1.37) | 0.71 | 0.95 (0.64-1.39) | 0.78 | 0.98 (0.66-1.46) | 0.93 |  |
| 5-6 per week | 41 | 9 | 0.65 (0.33-1.29) | 0.22 | 0.64 (0.32-1.28) | 0.21 | 0.64 (0.32-1.27) | 0.20 |  |
| At least once per day | 93 | 34 | 1.32 (0.87-2.01) | 0.20 | 1.35 (0.89-2.06) | 0.16 | 1.37 (0.90-2.10) | 0.14 |  |
| **Stone fruits (e.g. plum, apricot, peach, cherry)** |  |  |  |  |  |  |  |  |  |
| Never or <1 per month | 269 | 90 | 1.00 (1.00-1.00) | . | 1.00 (1.00-1.00) | . | 1.00 (1.00-1.00) | . | 0.94 |
| 1-3 per month | 175 | 57 | 0.90 (0.65-1.25) | 0.52 | 0.91 (0.66-1.27) | 0.59 | 0.90 (0.65-1.25) | 0.54 |  |
| Once a week | 140 | 50 | 1.12 (0.79-1.59) | 0.52 | 1.13 (0.80-1.59) | 0.50 | 1.08 (0.76-1.54) | 0.66 |  |
| 2-4 per week | 96 | 24 | 0.72 (0.46-1.13) | 0.15 | 0.72 (0.46-1.13) | 0.15 | 0.71 (0.44-1.13) | 0.15 |  |
| 5-6 per week | 20 | 6 | 0.77 (0.34-1.74) | 0.53 | 0.72 (0.31-1.66) | 0.43 | 0.66 (0.27-1.61) | 0.36 |  |
| At least once per day | 28 | 14 | 1.74 (0.98-3.08) | 0.06 | 1.71 (0.98-3.00) | 0.06 | 1.53 (0.90-2.62) | 0.12 |  |
| **Soft fruits (e.g. raspberry, strawberry, redcurrant, blackberry)** |  |  |  |  |  |  |  |  |  |
| Never or <1 per month | 265 | 89 | 1.00 (1.00-1.00) | . | 1.00 (1.00-1.00) | . | 1.00 (1.00-1.00) | . | 0.91 |
| 1-3 per month | 185 | 59 | 0.91 (0.66-1.26) | 0.58 | 0.96 (0.69-1.33) | 0.80 | 0.94 (0.67-1.32) | 0.73 |  |
| Once a week | 156 | 52 | 0.96 (0.69-1.35) | 0.83 | 0.98 (0.70-1.38) | 0.92 | 0.93 (0.65-1.31) | 0.67 |  |
| 2-4 per week | 93 | 30 | 0.97 (0.64-1.47) | 0.87 | 0.97 (0.64-1.48) | 0.90 | 0.98 (0.64-1.50) | 0.93 |  |
| 5-6 per week | 11 | 5 | 1.45 (0.56-3.74) | 0.44 | 1.32 (0.50-3.49) | 0.57 | 1.44 (0.50-4.19) | 0.50 |  |
| At least once per day | 18 | 6 | 0.90 (0.38-2.11) | 0.81 | 0.95 (0.41-2.20) | 0.91 | 1.08 (0.47-2.50) | 0.85 |  |
| **Fleshy fruits (e.g. apple, pear, banana, pineapple** |  |  |  |  |  |  |  |  |  |
| Never or <1 per month | 53 | 16 | 1.00 (1.00-1.00) | . | 1.00 (1.00-1.00) | . | 1.00 (1.00-1.00) | . | 0.80 |
| 1-3 per month | 49 | 14 | 0.94 (0.46-1.92) | 0.85 | 0.98 (0.47-2.03) | 0.95 | 0.84 (0.40-1.76) | 0.65 |  |
| Once a week | 106 | 37 | 1.19 (0.67-2.11) | 0.56 | 1.22 (0.68-2.17) | 0.51 | 1.10 (0.61-1.99) | 0.76 |  |
| 2-4 per week | 227 | 80 | 1.19 (0.70-2.02) | 0.52 | 1.22 (0.71-2.09) | 0.48 | 1.19 (0.68-2.07) | 0.55 |  |
| 5-6 per week | 89 | 28 | 1.04 (0.57-1.93) | 0.89 | 1.11 (0.59-2.06) | 0.75 | 1.11 (0.59-2.10) | 0.74 |  |
| At least once per day | 204 | 66 | 1.04 (0.61-1.78) | 0.89 | 1.08 (0.62-1.87) | 0.78 | 1.03 (0.59-1.81) | 0.91 |  |
| **Vine fruits (e.g. grape, melon, cantaloupe)** |  |  |  |  |  |  |  |  |  |
| Never or <1 per month | 251 | 89 | 1.00 (1.00-1.00) | . | 1.00 (1.00-1.00) | . | 1.00 (1.00-1.00) | . | 0.31 |
| 1-3 per month | 177 | 64 | 1.01 (0.73-1.39) | 0.97 | 1.03 (0.74-1.42) | 0.87 | 1.02 (0.73-1.42) | 0.92 |  |
| Once a week | 120 | 34 | 0.79 (0.53-1.17) | 0.23 | 0.82 (0.55-1.21) | 0.31 | 0.86 (0.58-1.28) | 0.46 |  |
| 2-4 per week | 108 | 31 | 0.78 (0.52-1.18) | 0.24 | 0.78 (0.52-1.18) | 0.24 | 0.80 (0.52-1.23) | 0.31 |  |
| 5-6 per week | 32 | 10 | 0.80 (0.42-1.52) | 0.49 | 0.76 (0.39-1.48) | 0.42 | 0.75 (0.39-1.46) | 0.40 |  |
| At least once per day | 40 | 13 | 0.94 (0.53-1.67) | 0.84 | 0.92 (0.52-1.63) | 0.78 | 1.00 (0.59-1.72) | 0.99 |  |

*Model 1 was unadjusted, **Model 2 was adjusted for age, sex, and smoking status, ***Model 3 was adjusted for age, sex, smoking status, and tumour stage, grade, size and multiplicity.

| **Table 3. Hazard ratios (95% confidence intervals) of pre-diagnosis vitamin supplement use and a first bladder cancer recurrence (n=633)** | | | | | | | | | |
| --- | --- | --- | --- | --- | --- | --- | --- | --- | --- |
|  |  |  | **Model 1*** |  | **Model 2**** |  | **Model 3***** |  |  |
|  | **n** | **events** | **HR (95% CI)** | **p-value** | **HR (95% CI)** | **p-value** | **HR (95% CI)** | **p-value** | **p trend** |
| **Vitamin supplement use** |  |  |  |  |  |  |  |  |  |
| Never used vitamins | 396 | 123 | 1.00 (1.00-1.00) | . | 1.00 (1.00-1.00) | . | 1.00 (1.00-1.00) | . | 0.93 |
| Used to use vitamins | 84 | 29 | 1.10 (0.74-1.64) | 0.64 | 1.09 (0.73-1.63) | 0.68 | 1.11 (0.73-1.68) | 0.63 |  |
| Use vitamins | 153 | 51 | 1.03 (0.74-1.43) | 0.86 | 1.01 (0.73-1.41) | 0.94 | 0.97 (0.70-1.35) | 0.86 |  |

| **Table 4. Hazard ratios (95% confidence intervals) of pre-diagnosis vegetable intake and multiple bladder cancer recurrences (n=728)** | | | | | | | | | |
| --- | --- | --- | --- | --- | --- | --- | --- | --- | --- |
|  |  |  | **Model 1*** |  | **Model 2**** |  | **Model 3***** |  |  |
|  | **n** | **events** | **HR (95% CI)** | **p-value** | **HR (95% CI)** | **p-value** | **HR (95% CI)** | **p-value** | **p trend** |
| **Fruit vegetables (e.g. tomato, cucumber, aubergine)** |  |  |  |  |  |  |  |  |  |
| Never or <1 per month | 44 | 25 | 1.00 (1.00-1.00) | . | 1.00 (1.00-1.00) | . | 1.00 (1.00-1.00) | . | 0.43 |
| 1-3 per month | 45 | 15 | 1.27 (0.85-1.90) | 0.25 | 1.24 (0.84-1.85) | 0.28 | 1.23 (0.84-1.81) | 0.29 |  |
| Once a week | 99 | 43 | 1.28 (1.04-1.57) | 0.02 | 1.25 (1.01-1.55) | 0.04 | 1.23 (1.00-1.51) | 0.05 |  |
| 2-4 per week | 269 | 151 | 1.18 (0.98-1.41) | 0.07 | 1.17 (0.97-1.42) | 0.10 | 1.14 (0.95-1.36) | 0.15 |  |
| 5-6 per week | 99 | 43 | 1.12 (0.93-1.36) | 0.23 | 1.13 (0.93-1.37) | 0.23 | 1.11 (0.92-1.34) | 0.26 |  |
| At least once per day | 172 | 114 | 1.23 (1.02-1.49) | 0.03 | 1.23 (1.01-1.50) | 0.04 | 1.19 (0.98-1.44) | 0.08 |  |
| **Flower vegetables (e.g. broccoli, cauliflower)** |  |  |  |  |  |  |  |  |  |
| Never or <1 per month | 68 | 39 | 1.00 (1.00-1.00) | . | 1.00 (1.00-1.00) | . | 1.00 (1.00-1.00) | . | 0.75 |
| 1-3 per month | 60 | 25 | 0.88 (0.74-1.04) | 0.12 | 0.89 (0.74-1.06) | 0.19 | 0.84 (0.69-1.01) | 0.07 |  |
| Once a week | 172 | 87 | 0.98 (0.82-1.15) | 0.77 | 0.98 (0.82-1.17) | 0.82 | 0.97 (0.81-1.16) | 0.76 |  |
| 2-4 per week | 323 | 162 | 1.05 (0.90-1.23) | 0.52 | 1.05 (0.89-1.24) | 0.57 | 1.03 (0.87-1.21) | 0.75 |  |
| 5-6 per week | 62 | 42 | 1.08 (0.89-1.32) | 0.41 | 1.10 (0.90-1.35) | 0.36 | 1.06 (0.87-1.30) | 0.56 |  |
| At least once per day | 43 | 36 | 0.84 (0.66-1.07) | 0.16 | 0.84 (0.65-1.09) | 0.19 | 0.81 (0.63-1.03) | 0.09 |  |
| **Leafy vegetables (e.g. spinach, cabbage, lettuce)** |  |  |  |  |  |  |  |  |  |
| Never or <1 per month | 67 | 31 | 1.00 (1.00-1.00) | . | 1.00 (1.00-1.00) | . | 1.00 (1.00-1.00) | . | 0.77 |
| 1-3 per month | 56 | 25 | 1.02 (0.81-1.29) | 0.86 | 1.04 (0.82-1.31) | 0.77 | 0.95 (0.74-1.21) | 0.68 |  |
| Once a week | 189 | 110 | 1.10 (0.88-1.37) | 0.39 | 1.12 (0.90-1.39) | 0.32 | 1.05 (0.85-1.30) | 0.66 |  |
| 2-4 per week | 312 | 154 | 1.09 (0.88-1.36) | 0.43 | 1.11 (0.89-1.39) | 0.36 | 1.06 (0.86-1.31) | 0.57 |  |
| 5-6 per week | 60 | 45 | 1.12 (0.89-1.40) | 0.34 | 1.14 (0.91-1.43) | 0.25 | 1.10 (0.89-1.36) | 0.38 |  |
| At least once per day | 44 | 26 | 0.97 (0.71-1.33) | 0.85 | 0.98 (0.72-1.33) | 0.87 | 0.94 (0.70-1.25) | 0.66 |  |
| **Stem vegetables (e.g. asparagus, celery, fennel)** |  |  |  |  |  |  |  |  |  |
| Never or <1 per month | 369 | 204 | 1.00 (1.00-1.00) | . | 1.00 (1.00-1.00) | . | 1.00 (1.00-1.00) | . | 0.77 |
| 1-3 per month | 169 | 79 | 1.11 (0.96-1.29) | 0.15 | 1.11 (0.96-1.29) | 0.17 | 1.07 (0.93-1.24) | 0.33 |  |
| Once a week | 105 | 56 | 1.04 (0.86-1.27) | 0.67 | 1.05 (0.86-1.28) | 0.64 | 1.05 (0.85-1.30) | 0.63 |  |
| 2-4 per week | 69 | 44 | 0.95 (0.81-1.12) | 0.54 | 0.96 (0.82-1.12) | 0.58 | 0.98 (0.84-1.15) | 0.82 |  |
| 5-6 per week | 6 | 3 | 0.91 (0.76-1.08) | 0.29 | 0.91 (0.77-1.07) | 0.25 | 0.88 (0.67-1.15) | 0.35 |  |
| At least once per day | 10 | 5 | 1.21 (0.78-1.86) | 0.40 | 1.13 (0.70-1.83) | 0.61 | 1.17 (0.68-2.01) | 0.58 |  |
| **Mushrooms** |  |  |  |  |  |  |  |  |  |
| Never or <1 per month | 177 | 90 | 1.00 (1.00-1.00) | . | 1.00 (1.00-1.00) | . | 1.00 (1.00-1.00) | . | 0.27 |
| 1-3 per month | 184 | 104 | 1.10 (0.95-1.27) | 0.20 | 1.12 (0.97-1.30) | 0.13 | 1.13 (0.98-1.32) | 0.10 |  |
| Once a week | 206 | 111 | 1.04 (0.91-1.20) | 0.53 | 1.06 (0.92-1.22) | 0.40 | 1.06 (0.93-1.21) | 0.35 |  |
| 2-4 per week | 143 | 71 | 1.07 (0.91-1.26) | 0.42 | 1.09 (0.93-1.29) | 0.30 | 1.10 (0.94-1.28) | 0.26 |  |
| 5-6 per week | 7 | 11 | 1.25 (0.91-1.73) | 0.17 | 1.34 (0.91-1.97) | 0.13 | 1.49 (0.99-2.25) | 0.06 |  |
| At least once per day | 11 | 4 | 1.14 (0.79-1.64) | 0.48 | 1.18 (0.81-1.70) | 0.39 | 1.09 (0.75-1.59) | 0.66 |  |

*Model 1 was unadjusted, **Model 2 was adjusted for age, sex, and smoking status, ***Model 3 was adjusted for age, sex, smoking status, and tumour stage, grade, size and multiplicity, and re-resection of a bladder tumour (second transurethral resection).

| **Table 4. (continued)** | | | | | | | | | |
| --- | --- | --- | --- | --- | --- | --- | --- | --- | --- |
|  |  |  | **Model 1*** |  | **Model 2**** |  | **Model 3***** |  |  |
|  | **n** | **events** | **HR (95% CI)** | **p-value** | **HR (95% CI)** | **p-value** | **HR (95% CI)** | **p-value** | **p trend** |
| **Bulbs (e.g. onion, garlic, leek, shallot)** |  |  |  |  |  |  |  |  |  |
| Never or <1 per month | 121 | 63 | 1.00 (1.00-1.00) | . | 1.00 (1.00-1.00) | . | 1.00 (1.00-1.00) | . | 0.54 |
| 1-3 per month | 103 | 50 | 0.98 (0.82-1.18) | 0.84 | 1.00 (0.84-1.20) | 1.00 | 0.97 (0.81-1.16) | 0.74 |  |
| Once a week | 170 | 108 | 0.97 (0.85-1.12) | 0.72 | 0.97 (0.84-1.12) | 0.68 | 0.95 (0.82-1.09) | 0.44 |  |
| 2-4 per week | 241 | 120 | 0.93 (0.81-1.08) | 0.34 | 0.93 (0.80-1.08) | 0.33 | 0.93 (0.81-1.07) | 0.31 |  |
| 5-6 per week | 59 | 29 | 1.25 (0.99-1.57) | 0.06 | 1.26 (1.00-1.58) | 0.05 | 1.25 (0.99-1.58) | 0.06 |  |
| At least once per day | 34 | 21 | 0.82 (0.64-1.04) | 0.10 | 0.80 (0.63-1.01) | 0.06 | 0.77 (0.61-0.97) | 0.03 |  |
| **Roots (e.g. beetroot, swede, carrot, parsnip)** |  |  |  |  |  |  |  |  |  |
| Never or <1 per month | 69 | 38 | 1.00 (1.00-1.00) | . | 1.00 (1.00-1.00) | . | 1.00 (1.00-1.00) | . | 0.12 |
| 1-3 per month | 71 | 44 | 0.86 (0.72-1.03) | 0.11 | 0.87 (0.72-1.06) | 0.16 | 0.83 (0.68-1.01) | 0.06 |  |
| Once a week | 173 | 62 | 0.90 (0.76-1.07) | 0.23 | 0.92 (0.77-1.10) | 0.34 | 0.93 (0.77-1.11) | 0.40 |  |
| 2-4 per week | 304 | 164 | 0.92 (0.79-1.09) | 0.34 | 0.92 (0.78-1.09) | 0.35 | 0.89 (0.76-1.05) | 0.17 |  |
| 5-6 per week | 68 | 54 | 0.93 (0.76-1.13) | 0.46 | 0.93 (0.76-1.14) | 0.48 | 0.92 (0.76-1.11) | 0.37 |  |
| At least once per day | 43 | 29 | 0.77 (0.60-0.99) | 0.04 | 0.76 (0.59-0.98) | 0.03 | 0.72 (0.56-0.93) | 0.01 |  |

*Model 1 was unadjusted, **Model 2 was adjusted for age, sex, and smoking status, ***Model 3 was adjusted for age, sex, smoking status, and tumour stage, grade, size and multiplicity, and re-resection of a bladder tumour (second transurethral resection).

| **Table 5. Hazard ratios (95% confidence intervals) of pre-diagnosis fruit intake and multiple bladder cancer recurrences (n=728)** | | | | | | | | | |
| --- | --- | --- | --- | --- | --- | --- | --- | --- | --- |
|  |  |  | **Model 1*** |  | **Model 2**** |  | **Model 3***** |  |  |
|  | **n** | **events** | **HR (95% CI)** | **p-value** | **HR (95% CI)** | **p-value** | **HR (95% CI)** | **p-value** | **p trend** |
| **Citrus fruits (e.g. orange, lemon, lime, grapefruit)** |  |  |  |  |  |  |  |  |  |
| Never or <1 per month | 223 | 118 | 1.00 (1.00-1.00) | . | 1.00 (1.00-1.00) | . | 1.00 (1.00-1.00) | . | 0.59 |
| 1-3 per month | 109 | 65 | 1.24 (1.04-1.48) | 0.02 | 1.25 (1.05-1.50) | 0.01 | 1.24 (1.04-1.46) | 0.01 |  |
| Once a week | 107 | 76 | 1.00 (0.87-1.16) | 0.95 | 1.01 (0.88-1.17) | 0.88 | 1.01 (0.87-1.17) | 0.92 |  |
| 2-4 per week | 155 | 72 | 1.02 (0.88-1.19) | 0.80 | 1.02 (0.87-1.19) | 0.80 | 1.03 (0.88-1.20) | 0.74 |  |
| 5-6 per week | 41 | 16 | 0.97 (0.80-1.19) | 0.80 | 0.97 (0.78-1.20) | 0.76 | 0.95 (0.76-1.20) | 0.68 |  |
| At least once per day | 93 | 44 | 1.14 (0.97-1.35) | 0.11 | 1.13 (0.96-1.34) | 0.15 | 1.14 (0.96-1.34) | 0.13 |  |
| **Stone fruits (e.g. plum, apricot, peach, cherry)** |  |  |  |  |  |  |  |  |  |
| Never or <1 per month | 269 | 146 | 1.00 (1.00-1.00) | . | 1.00 (1.00-1.00) | . | 1.00 (1.00-1.00) | . | 0.04 |
| 1-3 per month | 175 | 94 | 1.06 (0.93-1.20) | 0.37 | 1.05 (0.92-1.20) | 0.47 | 1.02 (0.90-1.17) | 0.72 |  |
| Once a week | 140 | 90 | 1.21 (1.04-1.42) | 0.02 | 1.21 (1.03-1.41) | 0.02 | 1.20 (1.02-1.41) | 0.03 |  |
| 2-4 per week | 96 | 35 | 0.99 (0.85-1.16) | 0.94 | 0.98 (0.83-1.16) | 0.84 | 0.98 (0.84-1.15) | 0.83 |  |
| 5-6 per week | 20 | 7 | 1.24 (0.75-2.04) | 0.40 | 1.22 (0.72-2.07) | 0.45 | 1.31 (0.82-2.11) | 0.26 |  |
| At least once per day | 28 | 19 | 1.23 (0.98-1.54) | 0.07 | 1.24 (0.99-1.54) | 0.06 | 1.25 (0.98-1.60) | 0.08 |  |
| **Soft fruits (e.g. raspberry, strawberry, redcurrant, blackberry)** |  |  |  |  |  |  |  |  |  |
| Never or <1 per month | 265 | 138 | 1.00 (1.00-1.00) | . | 1.00 (1.00-1.00) | . | 1.00 (1.00-1.00) | . | 0.88 |
| 1-3 per month | 185 | 105 | 0.93 (0.82-1.05) | 0.21 | 0.93 (0.82-1.05) | 0.22 | 0.94 (0.83-1.07) | 0.33 |  |
| Once a week | 156 | 80 | 1.04 (0.89-1.22) | 0.63 | 1.03 (0.88-1.21) | 0.70 | 1.03 (0.88-1.20) | 0.72 |  |
| 2-4 per week | 93 | 42 | 0.93 (0.79-1.10) | 0.38 | 0.94 (0.80-1.11) | 0.48 | 0.94 (0.79-1.11) | 0.46 |  |
| 5-6 per week | 11 | 16 | 0.96 (0.66-1.38) | 0.80 | 0.93 (0.64-1.34) | 0.69 | 1.01 (0.70-1.47) | 0.94 |  |
| At least once per day | 18 | 10 | 1.06 (0.81-1.38) | 0.69 | 1.07 (0.81-1.40) | 0.64 | 1.10 (0.81-1.50) | 0.53 |  |
| **Fleshy fruits (e.g. apple, pear, banana, pineapple** |  |  |  |  |  |  |  |  |  |
| Never or <1 per month | 53 | 25 | 1.00 (1.00-1.00) | . | 1.00 (1.00-1.00) | . | 1.00 (1.00-1.00) | . | 0.96 |
| 1-3 per month | 49 | 20 | 1.01 (0.79-1.29) | 0.95 | 1.02 (0.79-1.31) | 0.90 | 0.96 (0.72-1.27) | 0.77 |  |
| Once a week | 106 | 55 | 1.04 (0.84-1.29) | 0.70 | 1.06 (0.85-1.32) | 0.59 | 1.03 (0.82-1.29) | 0.83 |  |
| 2-4 per week | 227 | 136 | 1.03 (0.84-1.26) | 0.76 | 1.04 (0.85-1.29) | 0.69 | 1.01 (0.81-1.26) | 0.95 |  |
| 5-6 per week | 89 | 32 | 1.00 (0.81-1.25) | 0.98 | 1.01 (0.81-1.26) | 0.91 | 0.95 (0.75-1.21) | 0.67 |  |
| At least once per day | 204 | 123 | 1.03 (0.83-1.27) | 0.81 | 1.03 (0.83-1.28) | 0.79 | 1.00 (0.80-1.26) | 0.98 |  |
| **Vine fruits (e.g. grape, melon, cantaloupe)** |  |  |  |  |  |  |  |  |  |
| Never or <1 per month | 251 | 139 | 1.00 (1.00-1.00) | . | 1.00 (1.00-1.00) | . | 1.00 (1.00-1.00) | . | 0.83 |
| 1-3 per month | 177 | 105 | 1.09 (0.96-1.25) | 0.20 | 1.10 (0.96-1.25) | 0.18 | 1.11 (0.97-1.27) | 0.13 |  |
| Once a week | 120 | 53 | 0.94 (0.82-1.09) | 0.42 | 0.95 (0.83-1.10) | 0.51 | 0.97 (0.84-1.12) | 0.71 |  |
| 2-4 per week | 108 | 58 | 1.06 (0.88-1.27) | 0.56 | 1.06 (0.87-1.28) | 0.57 | 1.10 (0.90-1.35) | 0.36 |  |
| 5-6 per week | 32 | 17 | 1.01 (0.79-1.29) | 0.94 | 0.97 (0.76-1.24) | 0.83 | 0.98 (0.79-1.21) | 0.83 |  |
| At least once per day | 40 | 19 | 0.92 (0.70-1.20) | 0.53 | 0.94 (0.72-1.23) | 0.65 | 0.94 (0.73-1.22) | 0.65 |  |

*Model 1 was unadjusted, **Model 2 was adjusted for age, sex, and smoking status, ***Model 3 was adjusted for age, sex, smoking status, and tumour stage, grade, size and multiplicity, and re-resection of a bladder tumour (second transurethral resection).

| **Table 6. Hazard ratios (95% confidence intervals) of pre-diagnosis vitamin supplement use and multiple bladder cancer recurrences (n=633)** | | | | | | | | | |
| --- | --- | --- | --- | --- | --- | --- | --- | --- | --- |
|  |  |  | **Model 1*** |  | **Model 2**** |  | **Model 3***** |  |  |
|  | **n** | **events** | **HR (95% CI)** | **p-value** | **HR (95% CI)** | **p-value** | **HR (95% CI)** | **p-value** | **p trend** |
| **Vitamin supplement use** |  |  |  |  |  |  |  |  |  |
| Never used vitamins | 396 | 197 | 1.00 (1.00-1.00) |  | 1.00 (1.00-1.00) |  | 1.00 (1.00-1.00) |  | 0.12 |
| Used to use vitamins | 84 | 40 | 0.91 (0.72-1.16) | 0.46 | 0.89 (0.70-1.15) | 0.39 | 0.88 (0.68-1.13) | 0.32 |  |
| Use vitamins | 153 | 75 | 0.94 (0.83-1.06) | 0.32 | 0.92 (0.81-1.04) | 0.19 | 0.91 (0.80-1.04) | 0.16 |  |

**Post-diagnosis analysis**

| **Table 7. Hazard ratios (95% confidence intervals) of post-diagnosis vegetable intake and a first bladder cancer recurrence (n=389)** | | | | | | | | | |
| --- | --- | --- | --- | --- | --- | --- | --- | --- | --- |
|  |  |  | **Model 1*** |  | **Model 2**** |  | **Model 3***** |  |  |
|  | **n** | **events** | **HR (95% CI)** | **p-value** | **HR (95% CI)** | **p-value** | **HR (95% CI)** | **p-value** | **p trend** |
| **Fruit vegetables (e.g. tomato, cucumber, aubergine)** |  |  |  |  |  |  |  |  |  |
| Never or <1 per month | 28 | 13 | 1.00 (1.00-1.00) | . | 1.00 (1.00-1.00) | . | 1.00 (1.00-1.00) | . | 0.87 |
| 1-3 per month | 22 | 5 | 0.33 (0.12-0.88) | 0.03 | 0.30 (0.11-0.82) | 0.02 | 0.29 (0.11-0.80) | 0.02 |  |
| Once a week | 76 | 30 | 0.69 (0.35-1.36) | 0.29 | 0.67 (0.34-1.34) | 0.26 | 0.74 (0.37-1.45) | 0.38 |  |
| 2-4 per week | 169 | 61 | 0.61 (0.33-1.14) | 0.12 | 0.59 (0.31-1.11) | 0.10 | 0.70 (0.37-1.31) | 0.26 |  |
| 5-6 per week | 53 | 20 | 0.65 (0.31-1.36) | 0.25 | 0.65 (0.31-1.36) | 0.25 | 0.72 (0.34-1.53) | 0.39 |  |
| At least once per day | 41 | 15 | 0.60 (0.28-1.29) | 0.19 | 0.59 (0.27-1.28) | 0.18 | 0.78 (0.34-1.78) | 0.55 |  |
| **Flower vegetables (e.g. broccoli, cauliflower)** |  |  |  |  |  |  |  |  |  |
| Never or <1 per month | 39 | 16 | 1.00 (1.00-1.00) | . | 1.00 (1.00-1.00) | . | 1.00 (1.00-1.00) | . | 0.50 |
| 1-3 per month | 33 | 10 | 0.67 (0.31-1.45) | 0.31 | 0.65 (0.30-1.41) | 0.27 | 0.70 (0.33-1.50) | 0.36 |  |
| Once a week | 95 | 39 | 1.01 (0.57-1.81) | 0.97 | 0.99 (0.55-1.78) | 0.98 | 1.39 (0.78-2.48) | 0.26 |  |
| 2-4 per week | 187 | 63 | 0.74 (0.43-1.28) | 0.29 | 0.75 (0.43-1.31) | 0.31 | 1.00 (0.57-1.74) | 0.99 |  |
| 5-6 per week | 23 | 9 | 0.94 (0.41-2.15) | 0.89 | 0.92 (0.39-2.14) | 0.84 | 1.04 (0.43-2.56) | 0.92 |  |
| At least once per day | 12 | 7 | 1.47 (0.66-3.25) | 0.34 | 1.38 (0.61-3.10) | 0.44 | 1.94 (0.78-4.83) | 0.15 |  |
| **Leafy vegetables (e.g. spinach, cabbage, lettuce)** |  |  |  |  |  |  |  |  |  |
| Never or <1 per month | 34 | 15 | 1.00 (1.00-1.00) | . | 1.00 (1.00-1.00) | . | 1.00 (1.00-1.00) | . | 0.06 |
| 1-3 per month | 29 | 12 | 0.81 (0.39-1.65) | 0.55 | 0.79 (0.38-1.64) | 0.53 | 0.66 (0.32-1.35) | 0.26 |  |
| Once a week | 102 | 44 | 0.95 (0.52-1.70) | 0.85 | 0.93 (0.51-1.71) | 0.81 | 0.82 (0.46-1.46) | 0.50 |  |
| 2-4 per week | 191 | 61 | 0.63 (0.36-1.11) | 0.11 | 0.64 (0.36-1.12) | 0.12 | 0.51 (0.28-0.91) | 0.02 |  |
| 5-6 per week | 22 | 7 | 0.62 (0.25-1.52) | 0.29 | 0.62 (0.25-1.52) | 0.29 | 0.46 (0.17-1.27) | 0.13 |  |
| At least once per day | 11 | 5 | 0.95 (0.36-2.50) | 0.92 | 0.91 (0.34-2.43) | 0.85 | 0.83 (0.26-2.63) | 0.76 |  |
| **Stem vegetables (e.g. asparagus, celery, fennel)** |  |  |  |  |  |  |  |  |  |
| Never or <1 per month | 210 | 75 | 1.00 (1.00-1.00) | . | 1.00 (1.00-1.00) | . | 1.00 (1.00-1.00) | . | 0.17 |
| 1-3 per month | 68 | 25 | 1.03 (0.65-1.64) | 0.89 | 1.09 (0.68-1.73) | 0.73 | 1.21 (0.75-1.95) | 0.44 |  |
| Once a week | 57 | 20 | 0.90 (0.56-1.46) | 0.67 | 0.92 (0.57-1.49) | 0.73 | 1.11 (0.65-1.90) | 0.69 |  |
| 2-4 per week | 45 | 19 | 1.22 (0.73-2.04) | 0.44 | 1.26 (0.76-2.09) | 0.38 | 1.41 (0.83-2.40) | 0.20 |  |
| 5-6 per week | 6 | 3 | 1.25 (0.45-3.43) | 0.67 | 1.11 (0.39-3.19) | 0.84 | 1.02 (0.38-2.70) | 0.97 |  |
| At least once per day | 3 | 2 | 2.83 (0.49-16.30) | 0.24 | 2.64 (0.49-14.09) | 0.26 | 3.66 (0.67-19.90) | 0.13 |  |
| **Mushrooms** |  |  |  |  |  |  |  |  |  |
| Never or <1 per month | 73 | 24 | 1.00 (1.00-1.00) | . | 1.00 (1.00-1.00) | . | 1.00 (1.00-1.00) | . | 0.42 |
| 1-3 per month | 109 | 53 | 1.48 (0.91-2.39) | 0.11 | 1.46 (0.90-2.38) | 0.13 | 1.33 (0.81-2.17) | 0.26 |  |
| Once a week | 109 | 34 | 0.93 (0.54-1.58) | 0.78 | 0.95 (0.55-1.65) | 0.86 | 0.94 (0.54-1.64) | 0.82 |  |
| 2-4 per week | 93 | 33 | 1.02 (0.60-1.74) | 0.93 | 1.05 (0.61-1.79) | 0.87 | 1.08 (0.62-1.87) | 0.80 |  |
| 5-6 per week | 4 | 0 | 0.00 (0.00-0.00) | 0.00 | 0.00 (0.00-0.00) | 0.00 | 0.00 (0.00-0.00) | 0.00 |  |
| At least once per day | 1 | 0 | 0.00 (0.00-0.00) | 0.00 | 0.00 (0.00-0.00) | 0.00 | 0.00 (0.00-0.00) | 0.00 |  |

*Model 1 was unadjusted, **Model 2 was adjusted for age, sex, and smoking status, ***Model 3 was adjusted for age, sex, smoking status, and tumour stage, grade, size and multiplicity.

| **Table 7. (continued)** | | | | | | | | | |
| --- | --- | --- | --- | --- | --- | --- | --- | --- | --- |
|  |  |  | **Model 1*** |  | **Model 2**** |  | **Model 3***** |  |  |
|  | **n** | **events** | **HR (95% CI)** | **p-value** | **HR (95% CI)** | **p-value** | **HR (95% CI)** | **p-value** | **p trend** |
| **Bulbs (e.g. onion, garlic, leek, shallot)** |  |  |  |  |  |  |  |  |  |
| Never or <1 per month | 56 | 19 | 1.00 (1.00-1.00) | . | 1.00 (1.00-1.00) | . | 1.00 (1.00-1.00) | . | 0.10 |
| 1-3 per month | 44 | 22 | 1.49 (0.80-2.77) | 0.20 | 1.55 (0.83-2.87) | 0.17 | 1.58 (0.86-2.92) | 0.14 |  |
| Once a week | 103 | 41 | 1.18 (0.68-2.05) | 0.55 | 1.20 (0.69-2.08) | 0.53 | 1.14 (0.65-1.98) | 0.65 |  |
| 2-4 per week | 148 | 53 | 0.96 (0.57-1.64) | 0.89 | 0.99 (0.58-1.68) | 0.96 | 0.94 (0.55-1.62) | 0.82 |  |
| 5-6 per week | 29 | 6 | 0.50 (0.20-1.23) | 0.13 | 0.52 (0.21-1.27) | 0.15 | 0.48 (0.19-1.20) | 0.12 |  |
| At least once per day | 9 | 3 | 0.95 (0.28-3.19) | 0.93 | 0.99 (0.29-3.32) | 0.98 | 0.78 (0.20-3.11) | 0.73 |  |
| **Roots (e.g. beetroot, swede, carrot, parsnip)** |  |  |  |  |  |  |  |  |  |
| Never or <1 per month | 33 | 16 | 1.00 (1.00-1.00) | . | 1.00 (1.00-1.00) | . | 1.00 (1.00-1.00) | . | 0.01 |
| 1-3 per month | 42 | 19 | 0.80 (0.41-1.58) | 0.52 | 0.71 (0.35-1.44) | 0.35 | 0.81 (0.40-1.64) | 0.56 |  |
| Once a week | 97 | 37 | 0.60 (0.33-1.10) | 0.10 | 0.53 (0.28-1.00) | 0.05 | 0.54 (0.29-1.03) | 0.06 |  |
| 2-4 per week | 181 | 57 | 0.47 (0.26-0.84) | 0.01 | 0.43 (0.24-0.77) | 0.01 | 0.40 (0.22-0.76) | 0.00 |  |
| 5-6 per week | 27 | 12 | 0.71 (0.34-1.50) | 0.37 | 0.65 (0.30-1.38) | 0.26 | 0.62 (0.29-1.31) | 0.21 |  |
| At least once per day | 9 | 3 | 0.52 (0.15-1.77) | 0.29 | 0.44 (0.12-1.52) | 0.19 | 0.38 (0.11-1.36) | 0.14 |  |

*Model 1 was unadjusted, **Model 2 was adjusted for age, sex, and smoking status, ***Model 3 was adjusted for age, sex, smoking status, and tumour stage, grade, size and multiplicity.

| **Table 8. Hazard ratios (95% confidence intervals) of post-diagnosis fruit intake and a first bladder cancer recurrence (n=389)** | | | | | | | | | |
| --- | --- | --- | --- | --- | --- | --- | --- | --- | --- |
|  |  |  | **Model 1*** |  | **Model 2**** |  | **Model 3***** |  |  |
|  | **n** | **events** | **HR (95% CI)** | **p-value** | **HR (95% CI)** | **p-value** | **HR (95% CI)** | **p-value** | **p trend** |
| **Citrus fruits (e.g. orange, lemon, lime, grapefruit)** |  |  |  |  |  |  |  |  |  |
| Never or <1 per month | 146 | 56 | 1.00 (1.00-1.00) | . | 1.00 (1.00-1.00) | . | 1.00 (1.00-1.00) | . | 0.67 |
| 1-3 per month | 33 | 14 | 1.07 (0.62-1.87) | 0.80 | 1.13 (0.64-1.98) | 0.67 | 1.05 (0.61-1.80) | 0.86 |  |
| Once a week | 57 | 26 | 1.36 (0.85-2.18) | 0.20 | 1.44 (0.90-2.32) | 0.13 | 1.40 (0.85-2.29) | 0.18 |  |
| 2-4 per week | 85 | 25 | 0.76 (0.47-1.22) | 0.26 | 0.80 (0.49-1.30) | 0.37 | 0.88 (0.50-1.53) | 0.65 |  |
| 5-6 per week | 16 | 8 | 1.46 (0.70-3.06) | 0.31 | 1.65 (0.76-3.59) | 0.20 | 1.69 (0.74-3.84) | 0.21 |  |
| At least once per day | 52 | 15 | 0.69 (0.39-1.22) | 0.21 | 0.69 (0.39-1.21) | 0.19 | 0.74 (0.39-1.42) | 0.36 |  |
| **Stone fruits (e.g. plum, apricot, peach, cherry)** |  |  |  |  |  |  |  |  |  |
| Never or <1 per month | 185 | 76 | 1.00 (1.00-1.00) | . | 1.00 (1.00-1.00) | . | 1.00 (1.00-1.00) | . | 0.26 |
| 1-3 per month | 74 | 25 | 0.75 (0.48-1.18) | 0.22 | 0.76 (0.48-1.19) | 0.23 | 0.82 (0.52-1.30) | 0.41 |  |
| Once a week | 58 | 21 | 0.83 (0.51-1.35) | 0.46 | 0.85 (0.53-1.38) | 0.52 | 0.95 (0.57-1.57) | 0.83 |  |
| 2-4 per week | 45 | 13 | 0.63 (0.35-1.12) | 0.12 | 0.66 (0.37-1.17) | 0.16 | 0.74 (0.41-1.34) | 0.32 |  |
| 5-6 per week | 9 | 4 | 1.18 (0.46-3.03) | 0.74 | 1.23 (0.49-3.10) | 0.66 | 1.26 (0.47-3.42) | 0.65 |  |
| At least once per day | 18 | 5 | 0.56 (0.23-1.33) | 0.19 | 0.54 (0.23-1.25) | 0.15 | 0.56 (0.21-1.46) | 0.24 |  |
| **Soft fruits (e.g. raspberry, strawberry, redcurrant, blackberry)** |  |  |  |  |  |  |  |  |  |
| Never or <1 per month | 163 | 67 | 1.00 (1.00-1.00) | . | 1.00 (1.00-1.00) | . | 1.00 (1.00-1.00) | . | 0.20 |
| 1-3 per month | 92 | 32 | 0.78 (0.52-1.19) | 0.26 | 0.78 (0.51-1.19) | 0.24 | 0.79 (0.52-1.20) | 0.27 |  |
| Once a week | 83 | 29 | 0.79 (0.51-1.22) | 0.28 | 0.80 (0.52-1.24) | 0.32 | 0.84 (0.54-1.31) | 0.44 |  |
| 2-4 per week | 38 | 13 | 0.72 (0.41-1.29) | 0.27 | 0.74 (0.41-1.33) | 0.32 | 0.78 (0.42-1.45) | 0.43 |  |
| 5-6 per week | 5 | 2 | 1.14 (0.22-6.01) | 0.88 | 1.02 (0.19-5.39) | 0.98 | 1.28 (0.25-6.46) | 0.77 |  |
| At least once per day | 8 | 1 | 0.24 (0.03-1.71) | 0.15 | 0.23 (0.03-1.65) | 0.15 | 0.29 (0.04-2.08) | 0.22 |  |
| **Fleshy fruits (e.g. apple, pear, banana, pineapple** |  |  |  |  |  |  |  |  |  |
| Never or <1 per month | 29 | 11 | 1.00 (1.00-1.00) | . | 1.00 (1.00-1.00) | . | 1.00 (1.00-1.00) | . | 0.21 |
| 1-3 per month | 27 | 10 | 0.89 (0.39-2.07) | 0.79 | 0.90 (0.39-2.09) | 0.81 | 0.81 (0.33-1.97) | 0.64 |  |
| Once a week | 52 | 24 | 1.39 (0.67-2.90) | 0.38 | 1.44 (0.69-3.01) | 0.33 | 1.41 (0.64-3.14) | 0.40 |  |
| 2-4 per week | 126 | 51 | 1.01 (0.52-1.96) | 0.99 | 1.02 (0.52-1.99) | 0.95 | 0.96 (0.45-2.05) | 0.92 |  |
| 5-6 per week | 36 | 14 | 0.99 (0.44-2.23) | 0.98 | 1.04 (0.46-2.37) | 0.92 | 1.07 (0.43-2.67) | 0.89 |  |
| At least once per day | 119 | 34 | 0.66 (0.33-1.32) | 0.24 | 0.67 (0.33-1.36) | 0.27 | 0.67 (0.29-1.55) | 0.35 |  |
| **Vine fruits (e.g. grape, melon, cantaloupe)** |  |  |  |  |  |  |  |  |  |
| Never or <1 per month | 148 | 62 | 1.00 (1.00-1.00) | . | 1.00 (1.00-1.00) | . | 1.00 (1.00-1.00) | . | 0.07 |
| 1-3 per month | 78 | 31 | 0.91 (0.59-1.40) | 0.68 | 0.90 (0.59-1.39) | 0.65 | 0.90 (0.59-1.37) | 0.63 |  |
| Once a week | 68 | 22 | 0.67 (0.42-1.09) | 0.11 | 0.68 (0.42-1.11) | 0.12 | 0.68 (0.41-1.13) | 0.14 |  |
| 2-4 per week | 52 | 15 | 0.63 (0.35-1.11) | 0.11 | 0.63 (0.36-1.11) | 0.11 | 0.73 (0.39-1.36) | 0.32 |  |
| 5-6 per week | 18 | 9 | 1.24 (0.61-2.54) | 0.55 | 1.22 (0.60-2.50) | 0.58 | 1.34 (0.65-2.76) | 0.43 |  |
| At least once per day | 25 | 5 | 0.37 (0.16-0.85) | 0.02 | 0.36 (0.16-0.84) | 0.02 | 0.36 (0.15-0.87) | 0.02 |  |

*Model 1 was unadjusted, **Model 2 was adjusted for age, sex, and smoking status, ***Model 3 was adjusted for age, sex, smoking status, and tumour stage, grade, size and multiplicity.

| **Table 9. Hazard ratios (95% confidence intervals) of post-diagnosis vitamin supplement use and a first bladder cancer recurrence (n=284)** | | | | | | | | | |
| --- | --- | --- | --- | --- | --- | --- | --- | --- | --- |
|  |  |  | **Model 1*** |  | **Model 2**** |  | **Model 3***** |  |  |
|  | **n** | **events** | **HR (95% CI)** | **p-value** | **HR (95% CI)** | **p-value** | **HR (95% CI)** | **p-value** | **p trend** |
| **Vitamin supplement use** |  |  |  |  |  |  |  |  |  |
| Never used vitamins | 188 | 68 | 1.00 (1.00-1.00) | . | 1.00 (1.00-1.00) | . | 1.00 (1.00-1.00) | . | 0.96 |
| Used to use vitamins | 2 | 1 | 1.00 (1.00-1.00) | . | 1.00 (1.00-1.00) | . | 1.00 (1.00-1.00) | . |  |
| Use vitamins | 94 | 36 | 1.05 (0.70-1.57) | 0.81 | 1.04 (0.70-1.57) | 0.83 | 1.01 (0.66-1.53) | 0.97 |  |

| **Table 10. Hazard ratios (95% confidence intervals) of post-diagnosis vegetable intake and multiple bladder cancer recurrences (n=389)** | | | | | | | | | |
| --- | --- | --- | --- | --- | --- | --- | --- | --- | --- |
|  |  |  | **Model 1*** |  | **Model 2**** |  | **Model 3***** |  |  |
|  | **n** | **events** | **HR (95% CI)** | **p-value** | **HR (95% CI)** | **p-value** | **HR (95% CI)** | **p-value** | **p trend** |
| **Fruit vegetables (e.g. tomato, cucumber, aubergine)** |  |  |  |  |  |  |  |  |  |
| Never or <1 per month | 28 | 21 | 1.00 (1.00-1.00) | . | 1.00 (1.00-1.00) | . | 1.00 (1.00-1.00) | . | 0.21 |
| 1-3 per month | 22 | 8 | 0.76 (0.47-1.25) | 0.28 | 0.79 (0.47-1.32) | 0.37 | 0.84 (0.49-1.45) | 0.54 |  |
| Once a week | 76 | 45 | 0.97 (0.78-1.21) | 0.78 | 0.99 (0.79-1.25) | 0.97 | 1.03 (0.79-1.33) | 0.84 |  |
| 2-4 per week | 169 | 92 | 0.91 (0.75-1.10) | 0.33 | 0.93 (0.76-1.14) | 0.48 | 0.94 (0.75-1.19) | 0.62 |  |
| 5-6 per week | 53 | 28 | 0.94 (0.74-1.20) | 0.63 | 0.97 (0.75-1.24) | 0.78 | 0.97 (0.74-1.28) | 0.85 |  |
| At least once per day | 41 | 27 | 0.80 (0.62-1.03) | 0.08 | 0.81 (0.63-1.06) | 0.12 | 0.80 (0.61-1.06) | 0.12 |  |
| **Flower vegetables (e.g. broccoli, cauliflower)** |  |  |  |  |  |  |  |  |  |
| Never or <1 per month | 39 | 26 | 1.00 (1.00-1.00) | . | 1.00 (1.00-1.00) | . | 1.00 (1.00-1.00) | . | 0.46 |
| 1-3 per month | 33 | 19 | 1.08 (0.85-1.36) | 0.52 | 1.10 (0.87-1.40) | 0.42 | 1.09 (0.86-1.38) | 0.47 |  |
| Once a week | 95 | 63 | 1.01 (0.85-1.21) | 0.88 | 1.06 (0.87-1.28) | 0.57 | 1.06 (0.85-1.34) | 0.59 |  |
| 2-4 per week | 187 | 90 | 1.04 (0.87-1.24) | 0.66 | 1.08 (0.89-1.31) | 0.43 | 1.05 (0.85-1.30) | 0.63 |  |
| 5-6 per week | 23 | 12 | 1.15 (0.87-1.52) | 0.32 | 1.21 (0.91-1.60) | 0.20 | 1.12 (0.79-1.60) | 0.53 |  |
| At least once per day | 12 | 11 | 0.75 (0.58-0.96) | 0.02 | 0.80 (0.60-1.05) | 0.11 | 0.72 (0.51-1.02) | 0.07 |  |
| **Leafy vegetables (e.g. spinach, cabbage, lettuce)** |  |  |  |  |  |  |  |  |  |
| Never or <1 per month | 34 | 19 | 1.00 (1.00-1.00) | . | 1.00 (1.00-1.00) | . | 1.00 (1.00-1.00) | . | 0.46 |
| 1-3 per month | 29 | 24 | 0.94 (0.70-1.28) | 0.70 | 0.95 (0.70-1.29) | 0.74 | 0.93 (0.68-1.29) | 0.67 |  |
| Once a week | 102 | 70 | 0.95 (0.74-1.22) | 0.67 | 0.96 (0.74-1.23) | 0.72 | 0.96 (0.72-1.27) | 0.75 |  |
| 2-4 per week | 191 | 90 | 0.95 (0.74-1.21) | 0.67 | 0.96 (0.75-1.23) | 0.74 | 0.95 (0.70-1.27) | 0.71 |  |
| 5-6 per week | 22 | 9 | 1.02 (0.74-1.42) | 0.88 | 1.04 (0.74-1.45) | 0.82 | 0.98 (0.67-1.43) | 0.91 |  |
| At least once per day | 11 | 9 | 0.66 (0.47-0.92) | 0.01 | 0.68 (0.48-0.95) | 0.03 | 0.66 (0.44-1.00) | 0.05 |  |
| **Stem vegetables (e.g. asparagus, celery, fennel)** |  |  |  |  |  |  |  |  |  |
| Never or <1 per month | 210 | 130 | 1.00 (1.00-1.00) | . | 1.00 (1.00-1.00) | . | 1.00 (1.00-1.00) | . | 0.13 |
| 1-3 per month | 68 | 29 | 0.98 (0.84-1.14) | 0.80 | 0.99 (0.85-1.16) | 0.93 | 0.98 (0.84-1.16) | 0.85 |  |
| Once a week | 57 | 29 | 1.12 (0.90-1.39) | 0.30 | 1.11 (0.90-1.38) | 0.33 | 1.16 (0.92-1.45) | 0.21 |  |
| 2-4 per week | 45 | 26 | 1.05 (0.85-1.29) | 0.64 | 1.07 (0.86-1.31) | 0.55 | 1.14 (0.91-1.43) | 0.25 |  |
| 5-6 per week | 6 | 4 | 0.86 (0.59-1.26) | 0.44 | 0.89 (0.60-1.31) | 0.56 | 0.92 (0.64-1.32) | 0.66 |  |
| At least once per day | 3 | 3 | 1.46 (1.24-1.72) | 0.00 | 1.52 (1.25-1.86) | 0.00 | 1.39 (1.04-1.87) | 0.03 |  |
| **Mushrooms** |  |  |  |  |  |  |  |  |  |
| Never or <1 per month | 73 | 35 | 1.00 (1.00-1.00) | . | 1.00 (1.00-1.00) | . | 1.00 (1.00-1.00) | . | 0.38 |
| 1-3 per month | 109 | 89 | 1.05 (0.88-1.26) | 0.59 | 1.06 (0.88-1.27) | 0.55 | 1.03 (0.85-1.24) | 0.79 |  |
| Once a week | 109 | 51 | 1.19 (0.97-1.46) | 0.09 | 1.18 (0.95-1.47) | 0.13 | 1.19 (0.95-1.48) | 0.13 |  |
| 2-4 per week | 93 | 46 | 1.05 (0.86-1.28) | 0.61 | 1.04 (0.85-1.27) | 0.70 | 1.04 (0.85-1.27) | 0.72 |  |
| 5-6 per week | 4 | 0 | 1.00 (1.00-1.00) | . | 1.00 (1.00-1.00) | . | 1.00 (1.00-1.00) | . |  |
| At least once per day | 1 | 0 | 1.00 (1.00-1.00) | . | 1.00 (1.00-1.00) | . | 1.00 (1.00-1.00) | . |  |

*Model 1 was unadjusted, **Model 2 was adjusted for age, sex, and smoking status, ***Model 3 was adjusted for age, sex, smoking status, and tumour stage, grade, size and multiplicity, and re-resection of a bladder tumour (second transurethral resection).

| **Table 10. (continued)** | | | | | | | | | |
| --- | --- | --- | --- | --- | --- | --- | --- | --- | --- |
|  |  |  | **Model 1*** |  | **Model 2**** |  | **Model 3***** |  |  |
|  | **n** | **events** | **HR (95% CI)** | **p-value** | **HR (95% CI)** | **p-value** | **HR (95% CI)** | **p-value** | **p trend** |
| **Bulbs (e.g. onion, garlic, leek, shallot)** |  |  |  |  |  |  |  |  |  |
| Never or <1 per month | 56 | 29 | 1.00 (1.00-1.00) | . | 1.00 (1.00-1.00) | . | 1.00 (1.00-1.00) | . | 0.06 |
| 1-3 per month | 44 | 34 | 0.96 (0.75-1.24) | 0.77 | 0.97 (0.75-1.25) | 0.81 | 0.99 (0.76-1.29) | 0.95 |  |
| Once a week | 103 | 58 | 1.05 (0.83-1.32) | 0.69 | 1.04 (0.83-1.31) | 0.72 | 1.04 (0.83-1.30) | 0.76 |  |
| 2-4 per week | 148 | 86 | 0.88 (0.71-1.09) | 0.25 | 0.88 (0.71-1.10) | 0.26 | 0.88 (0.71-1.09) | 0.24 |  |
| 5-6 per week | 29 | 6 | 1.10 (0.75-1.60) | 0.64 | 1.08 (0.73-1.60) | 0.69 | 0.89 (0.58-1.37) | 0.61 |  |
| At least once per day | 9 | 8 | 0.67 (0.47-0.95) | 0.03 | 0.67 (0.46-0.96) | 0.03 | 0.61 (0.38-0.97) | 0.03 |  |
| **Roots (e.g. beetroot, swede, carrot, parsnip)** |  |  |  |  |  |  |  |  |  |
| Never or <1 per month | 33 | 26 | 1.00 (1.00-1.00) | . | 1.00 (1.00-1.00) | . | 1.00 (1.00-1.00) | . | 0.19 |
| 1-3 per month | 42 | 30 | 0.75 (0.59-0.95) | 0.02 | 0.75 (0.59-0.96) | 0.02 | 0.81 (0.62-1.06) | 0.12 |  |
| Once a week | 97 | 57 | 0.86 (0.68-1.09) | 0.21 | 0.88 (0.69-1.12) | 0.30 | 0.89 (0.70-1.14) | 0.36 |  |
| 2-4 per week | 181 | 89 | 0.83 (0.67-1.02) | 0.07 | 0.85 (0.68-1.06) | 0.14 | 0.85 (0.68-1.06) | 0.15 |  |
| 5-6 per week | 27 | 14 | 0.79 (0.59-1.05) | 0.11 | 0.80 (0.58-1.09) | 0.16 | 0.80 (0.56-1.13) | 0.20 |  |
| At least once per day | 9 | 5 | 0.67 (0.36-1.23) | 0.19 | 0.69 (0.37-1.28) | 0.23 | 0.65 (0.34-1.26) | 0.20 |  |

*Model 1 was unadjusted, **Model 2 was adjusted for age, sex, and smoking status, ***Model 3 was adjusted for age, sex, smoking status, and tumour stage, grade, size and multiplicity, and re-resection of a bladder tumour (second transurethral resection).

| **Table 11. Hazard ratios (95% confidence intervals) of post-diagnosis fruit intake and multiple bladder cancer recurrences (n=389)** | | | | | | | | | |
| --- | --- | --- | --- | --- | --- | --- | --- | --- | --- |
|  |  |  | **Model 1*** |  | **Model 2**** |  | **Model 3***** |  |  |
|  | **n** | **events** | **HR (95% CI)** | **p-value** | **HR (95% CI)** | **p-value** | **HR (95% CI)** | **p-value** | **p trend** |
| **Citrus fruits (e.g. orange, lemon, lime, grapefruit)** |  |  |  |  |  |  |  |  |  |
| Never or <1 per month | 146 |  | 1.00 (1.00-1.00) | . | 1.00 (1.00-1.00) | . | 1.00 (1.00-1.00) | . | 0.65 |
| 1-3 per month | 33 |  | 0.89 (0.75-1.06) | 0.18 | 0.86 (0.72-1.04) | 0.12 | 0.85 (0.71-1.01) | 0.07 |  |
| Once a week | 57 |  | 0.93 (0.79-1.10) | 0.39 | 0.92 (0.78-1.09) | 0.33 | 0.85 (0.72-1.01) | 0.07 |  |
| 2-4 per week | 85 |  | 1.03 (0.87-1.22) | 0.74 | 1.05 (0.89-1.23) | 0.57 | 1.05 (0.90-1.22) | 0.51 |  |
| 5-6 per week | 16 |  | 1.01 (0.78-1.31) | 0.92 | 0.99 (0.77-1.28) | 0.96 | 0.93 (0.70-1.23) | 0.61 |  |
| At least once per day | 52 |  | 0.91 (0.70-1.17) | 0.45 | 0.91 (0.70-1.18) | 0.46 | 0.88 (0.65-1.19) | 0.40 |  |
| **Stone fruits (e.g. plum, apricot, peach, cherry)** |  |  |  |  |  |  |  |  |  |
| Never or <1 per month | 185 |  | 1.00 (1.00-1.00) | . | 1.00 (1.00-1.00) | . | 1.00 (1.00-1.00) | . | 0.67 |
| 1-3 per month | 74 |  | 0.97 (0.82-1.15) | 0.70 | 0.98 (0.82-1.16) | 0.80 | 0.99 (0.82-1.19) | 0.89 |  |
| Once a week | 58 |  | 0.91 (0.74-1.11) | 0.34 | 0.91 (0.74-1.13) | 0.41 | 0.91 (0.74-1.12) | 0.39 |  |
| 2-4 per week | 45 |  | 0.95 (0.79-1.15) | 0.61 | 0.96 (0.79-1.16) | 0.66 | 0.97 (0.79-1.19) | 0.74 |  |
| 5-6 per week | 9 |  | 1.08 (0.78-1.50) | 0.65 | 1.09 (0.81-1.47) | 0.57 | 1.09 (0.80-1.49) | 0.59 |  |
| At least once per day | 18 |  | 0.90 (0.73-1.12) | 0.34 | 0.94 (0.75-1.17) | 0.56 | 0.98 (0.78-1.24) | 0.87 |  |
| **Soft fruits (e.g. raspberry, strawberry, redcurrant, blackberry)** |  |  |  |  |  |  |  |  |  |
| Never or <1 per month | 163 |  | 1.00 (1.00-1.00) | . | 1.00 (1.00-1.00) | . | 1.00 (1.00-1.00) | . | 0.32 |
| 1-3 per month | 92 |  | 0.97 (0.82-1.14) | 0.72 | 0.99 (0.83-1.17) | 0.89 | 0.93 (0.79-1.11) | 0.43 |  |
| Once a week | 83 |  | 0.92 (0.78-1.08) | 0.31 | 0.93 (0.79-1.09) | 0.38 | 0.90 (0.77-1.05) | 0.19 |  |
| 2-4 per week | 38 |  | 0.78 (0.66-0.91) | 0.00 | 0.79 (0.67-0.92) | 0.00 | 0.76 (0.63-0.92) | 0.00 |  |
| 5-6 per week | 5 |  | 1.50 (0.92-2.46) | 0.10 | 1.53 (0.95-2.48) | 0.08 | 1.58 (0.98-2.54) | 0.06 |  |
| At least once per day | 8 |  | 1.38 (0.90-2.12) | 0.13 | 1.36 (0.89-2.09) | 0.16 | 1.36 (0.86-2.16) | 0.19 |  |
| **Fleshy fruits (e.g. apple, pear, banana, pineapple** |  |  |  |  |  |  |  |  |  |
| Never or <1 per month | 29 |  | 1.00 (1.00-1.00) | . | 1.00 (1.00-1.00) | . | 1.00 (1.00-1.00) | . | 0.19 |
| 1-3 per month | 27 |  | 0.87 (0.66-1.14) | 0.31 | 0.90 (0.68-1.18) | 0.44 | 0.76 (0.57-1.00) | 0.05 |  |
| Once a week | 52 |  | 0.85 (0.66-1.10) | 0.21 | 0.85 (0.65-1.11) | 0.23 | 0.76 (0.58-0.99) | 0.04 |  |
| 2-4 per week | 126 |  | 0.85 (0.68-1.08) | 0.18 | 0.87 (0.69-1.09) | 0.23 | 0.80 (0.62-1.02) | 0.08 |  |
| 5-6 per week | 36 |  | 0.90 (0.70-1.16) | 0.43 | 0.91 (0.71-1.16) | 0.45 | 0.81 (0.62-1.05) | 0.11 |  |
| At least once per day | 119 |  | 0.81 (0.64-1.03) | 0.08 | 0.83 (0.66-1.05) | 0.12 | 0.75 (0.59-0.97) | 0.03 |  |
| **Vine fruits (e.g. grape, melon, cantaloupe)** |  |  |  |  |  |  |  |  |  |
| Never or <1 per month | 148 |  | 1.00 (1.00-1.00) | . | 1.00 (1.00-1.00) | . | 1.00 (1.00-1.00) | . | 0.81 |
| 1-3 per month | 78 |  | 0.99 (0.86-1.15) | 0.93 | 0.99 (0.86-1.15) | 0.94 | 0.95 (0.82-1.10) | 0.50 |  |
| Once a week | 68 |  | 1.01 (0.83-1.23) | 0.90 | 1.02 (0.84-1.25) | 0.82 | 0.99 (0.81-1.20) | 0.89 |  |
| 2-4 per week | 52 |  | 0.98 (0.81-1.19) | 0.83 | 0.99 (0.81-1.22) | 0.94 | 1.03 (0.82-1.29) | 0.80 |  |
| 5-6 per week | 18 |  | 1.30 (1.00-1.70) | 0.05 | 1.34 (1.02-1.76) | 0.04 | 1.38 (1.06-1.80) | 0.02 |  |
| At least once per day | 25 |  | 0.74 (0.58-0.95) | 0.02 | 0.76 (0.60-0.97) | 0.03 | 0.72 (0.55-0.96) | 0.03 |  |

*Model 1 was unadjusted, **Model 2 was adjusted for age, sex, and smoking status, ***Model 3 was adjusted for age, sex, smoking status, and tumour stage, grade, size and multiplicity, and re-resection of a bladder tumour (second transurethral resection).

| **Table 12. Hazard ratios (95% confidence intervals) of post-diagnosis vitamin supplement use and multiple bladder cancer recurrences (n=284)** | | | | | | | | | |
| --- | --- | --- | --- | --- | --- | --- | --- | --- | --- |
|  |  |  | **Model 1*** |  | **Model 2**** |  | **Model 3***** |  |  |
|  | **n** | **events** | **HR (95% CI)** | **p-value** | **HR (95% CI)** | **p-value** | **HR (95% CI)** | **p-value** | **p trend** |
| **Vitamin supplement use** |  |  |  |  |  |  |  |  |  |
| Never used vitamins | 188 | 105 | 1.00 (1.00-1.00) |  | 1.00 (1.00-1.00) |  | 1.00 (1.00-1.00) |  | 0.40 |
| Used to use vitamins | 2 | 1 | 1.00 (1.00-1.00) | . | 1.00 (1.00-1.00) | . | 1.00 (1.00-1.00) | . |  |
| Use vitamins | 94 | 53 | 0.96 (0.84-1.11) | 0.61 | 0.98 (0.84-1.13) | 0.77 | 0.95 (0.83-1.09) | 0.43 |  |
